# Delayed Cutaneous Wound Healing and Aberrant Expression of Hair Follicle Stem Cell Markers in Mice Selectively Lacking Ctip2 in Epidermis

**DOI:** 10.1371/journal.pone.0029999

**Published:** 2012-02-21

**Authors:** Xiaobo Liang, Shreya Bhattacharya, Gaurav Bajaj, Gunjan Guha, Zhixing Wang, Hyo-Sang Jang, Mark Leid, Arup Kumar Indra, Gitali Ganguli-Indra

**Affiliations:** 1 Department of Pharmaceutical Sciences, College of Pharmacy, Oregon State University, Corvallis, United States of America; 2 Molecular and Cell Biology Program, Oregon State University, Corvallis, Oregon, United States of America; 3 Environmental Health Science Centre, Oregon State University, Corvallis, Oregon, United States of America; 4 Department of Dermatology, Oregon Health and Science University, Portland, Oregon, United States of America; University of Munich, Germany

## Abstract

**Background:**

COUP-TF interacting protein 2 [(Ctip2), also known as Bcl11b] is an important regulator of skin homeostasis, and is overexpressed in head and neck cancer. Ctip2^ep−/−^ mice, selectively ablated for Ctip2 in epidermal keratinocytes, exhibited impaired terminal differentiation and delayed epidermal permeability barrier (EPB) establishment during development, similar to what was observed in Ctip2 null (Ctip2**^−/−^**) mice. Considering that as an important role of Ctip2, and the fact that molecular networks which underlie cancer progression partially overlap with those responsible for tissue remodeling, we sought to determine the role of Ctip2 during cutaneous wound healing.

**Methodology/Principal Findings:**

Full thickness excisional wound healing experiments were performed on Ctip2^L2/L2^ and Ctip2^ep−/−^ animals per time point and used for harvesting samples for histology, immunohistochemistry (IHC) and immunoblotting. Results demonstrated inherent defects in proliferation and migration of Ctip2 lacking keratinocytes during re-epithelialization. Mutant mice exhibited reduced epidermal proliferation, delayed keratinocyte activation, altered cell-cell adhesion and impaired ECM development. Post wounding, Ctip2^ep−/−^ mice wounds displayed lack of E-Cadherin suppression in the migratory tongue, insufficient expression of alpha smooth muscle actin (alpha SMA) in the dermis, and robust induction of K8. Importantly, dysregulated expression of several hair follicle (HF) stem cell markers such as K15, NFATc1, CD133, CD34 and Lrig1 was observed in mutant skin during wound repair.

**Conclusions/Significance:**

Results confirm a cell autonomous role of keratinocytic Ctip2 to modulate cell migration, proliferation and/or differentiation, and to maintain HF stem cells during cutaneous wounding. Furthermore, Ctip2 in a non-cell autonomous manner regulated granulation tissue formation and tissue contraction during wound closure.

## Introduction

Cutaneous wound healing is a highly coordinated physiological process which involves a cross-talk between different cell types such as keratinocytes, fibroblasts, and immune cells [1], [2], [3], [4]. Upon injury there is a break in EPB function, and regeneration of the epidermis post wounding involves activation, migration and proliferation of keratinocytes from surrounding epidermis and adnexal structures (HF and sweat gland) [5], [6]. Re-epithelialization after epidermal injury involves resurfacing of the wound with new epithelium thereby providing rapid restoration of epidermal integrity and barrier function [7], [8], [9]. The changes in classic Ca ^2+^ -dependent cell–cell adhesion molecules such as E-and/or P-cadherin also play distinct roles in supply of keratinocytes toward a wound re-epithelialization [10], [11]. Wound repair occurs in the proliferative phase where fibroblasts provide the collagen framework for dermal regeneration, and pericytes and endothelial cells together participate in regeneration of the outer layer of capillaries in the angiogenic process. Migration and proliferation at the periphery of the wound are regulated by various growth factors, integrins, the extracellular matrix, and other regulatory proteins [8], [12].

The mitotically active basal layer of the skin express Keratin 5 (K5) and K14 and the differentiated keratinocytes express K1 and K10 [13], [14]. The activated suprabasal keratinocytes in wound healing, hyperproliferative diseases such as psoriasis and cancer express K6, K16 and K17 [14], [15], [16]. K6 is widely expressed at the wound edge and over the wound bed [14], [17], [18], [19]. In mouse skin, K16 is particularly involved in the re-epithelialization process by affecting migration of keratincoytes [16], [20]. K8 and K18 are the first keratins expressed during embryogenesis but not in the adult epidermis [7]. Invasive growth and malignancy of both human and murine epithelial tumors are linked with increased levels of K8 [21]. Increased expression of K8 has also been linked to a reduced re-epithelialization efficiency at wound margins [7]. K15, intermediate filament protein, is expressed mainly in the basal keratinocytes of stratified tissues or slowly turning over basal cells and also in subset of keratinocytes in the outer root sheath of HF [22]. The expression of K15 protein is downregulated in activated keratinocytes of hyperproliferating epidermis such as in wound bed, psoriasis and hypertrophic scars [4], [23].

Epithelialization during wound repair is mainly carried out by keratinocytes, which play an important role during this process. After the wound surface is covered by keratinocytes, expression of integrins and basal keratins by suprabasal cells decreases, leading to terminal differentiation in the outer layers of unwounded epidermis [8]. Cutaneous stem cells within the undamaged adult epidermis reside in the bulge region of the HF, keratinocytes of the interfollicular epidermis (IFE) and sebaceous glands [24], [25], [26], [27]. Skin wound repair and regeneration after wounding depends on the long-lived stem cells in the IFE and HF to contribute to re-epithelialization of wounds in vivo [5], [24], [28], [29], [30]. Epithelial stem cell marker K15 is known to be expressed preferentially in stable or in basal cells that turn over very slowly, and is more tightly coupled to a mature basal keratinocyte phenotype [22]. HF stem cell markers such as Nuclear factor of activated T-cells, cytoplasmic 1 (NFATc1), CD34 and Prominin-1/CD133 are all known to contribute in regulating epidermal stem cell quiescence, location, proliferation, wound healing and in tumor formation [31], [32], [33], [34], [35], [36], [37]. The stem/progenitor cell marker CD133 is expressed in the specialized mesenchymal cells at the base of the HF in normal skin [34], [38]. K15 and stem/progenitor cell marker CD133/prominin-1 (CD133) is strongly expressed during cell expansion of cultured human keratinocytes from skin explants [39]. 12-*O*-tetradecanoylphorbol-13-acetate (TPA)-induced HF stem cell activation and tumor formation in mice requires CD34. NFATc1 is expressed exclusively in the bulge region of the HF and both gain- and loss-of function studies demonstrate an inhibitory role for NFATc1 in stem cell activation in the HF [33].

Chicken ovalbumin upstream promoter transcription factor (COUP-TF) interacting protein 2 [(Ctip2) is a C2H2 zing finger transcriptional regulatory protein that regulates transcription by direct DNA binding activity or by interacting with COUP-TF nuclear receptor proteins [40], [41]. Ctip2 is highly expressed in mouse skin during development as well as in adult skin [42], [43]. Our lab has previously shown that Ctip2 controls epidermal proliferation/differentiation, formation of EPB, and is a key regulator of expression of subset of genes involved in those processes [44]. In a recent study we have shown that CTIP2 expression is upregulated in human head and neck tumors and in atopic dermatitis [45], [46]. Interestingly, Ctip2^ep−/−^ mice, selectively ablated for Ctip2 in epidermal keratinocytes, exhibited impaired terminal differentiation and delayed EPB establishment during development, similar to what was observed in Ctip2 null (Ctip2**^−/−^**) mice. Considering that as an important role of Ctip2, we herein examined the role of Ctip2 in wound healing process. In the present study, we report that, wound healing in mice with Ctip2 deletion in the epidermis is delayed due to delayed re-epithelialization, decreased proliferation and altered expression of HF stem cell markers. The delay in re-epithelialization is possibly due to tightly packed epidermis caused by inhibition of loss of cell-cell adhesion due to elevated E-cadherin expression in the wound adjacent mutant epidermis.

## Materials and Methods

### Cell culture

Primary keratinocytes were isolated and cultured using a modified version of a previously described protocol [47]. Briefly, a piece of dorsal newborn mice skin was incubated in 2 mg/ml dispase overnight at 4°C. The epidermal layer was separated from the dermal layer and incubated in TrypLE Select (Invitrogen, Carlsbad, CA) for 20 min at RT. Cells were rinsed and plated at a density of 1.5×10^5^ cells/cm^2^ in keratinocyte culture medium KCM (EMEM supplemented with 8% chelex-FBS, 10 ng/ml EGF, 0.05 mM CaCl_2_, 1× antibiotic and antimicotic). Cells were grown at 35°C in a humidified 5% CO_2_ incubator and medium was changed every 2∼3 days.

### *In vitro* scratch assay

An *in vitro* scratch wound healing assay was done as described earlier with a slight modification [48]. Briefly, primary mouse keratinocytes from Ctip2-null mice were grown to about 60∼70% confluence in KCM and part of cells were removed by scratching the layer with pipette tips. After rinsing detached cells off with PBS and KCM, cells were incubated at 35°C in a humidified 5% CO_2_ incubator. Images were captured everyday for 4 days after wounding using a CCD digital camera MicroPublisher 5.0 (QImaging, Surrey, Canada) connected to a phase contrast microscope Axiovert 40CFL (Carl Zeiss, Thornwood, NY). Migration of cells was quantified by measuring and averaging the distances between the borders of cells at 200 µm intervals in each captured image using AxioVision version 4.6 (Carl Zeiss) software. Nine regions were analyzed in each well at each time point and each genotype, and the result was expressed as the mean ± SEM. All experiments were performed in the presence of 1 µg or 5 µg/ml of mitomycin-C to exclude any non-migration contributors such as increased cell number.

### TPA-induced proliferation

Eight-weeks old C57BL6 wild type male mice (*n* = 3) were shaved thoroughly on the dorsal side. 24 h later, the shaved skin was topically treated with 10 nmol TPA (Sigma: St.Louis, MO, USA) in 100 µl 70% ethanol or 70% ethanol alone (vehicle control) three times every 48 h. After 24 h following the last treatment, skin was isolated. A portion of the collected skin was fixed in 4% paraformaldehyde and embedded in paraffin for histological staining and IHC study. Another portion of the skin sample was used for Western blot analysis [49].

### Retinoic Acid (RA)-induced proliferation

Eight-week old male C57BL6 wild type mice (*n* = 3) were shaved dorsally and after 24 h, treated four times every 24 h with 40 nmol RA [(in 100 µl 70% ethanol),] or 70% ethanol alone (vehicle control). 24 h after the final treatment, the treated skin was isolated for histological and IHC analysis (fixed in 4% paraformaldehye and embedded in paraffin), and for Western blotting.

### Hair cycle stages

Induction of hair growth by depilation and harvesting skin samples: In order to synchronize hair cycling, wax depilation was performed on 6–8-weeks old male C57BL/6 mice on the dorsal skin as described [50], [51]. Post depilation, mice were at telogen stage of HF development as confirmed by pink color of the skin (Day 0), anagen at Day 3, confirmed by skin darkening due to follicular melanogenesis, catagen at Day 16 and telogene at Day 25 [50], [52], [53]. The hair on the back of the mouse was carefully shaved using an electric razor 1 day before taking the skin samples to allow easier tissue sectioning and observation. The tissue samples were processed and sectioned to perform Hematoxylin & Eosin (H&E) staining and IHC [46].

Normal hair cycle: Skin samples from normal hair cycle stages were collected at post natal days 28 (2^nd^ anagen) and 49 (2^nd^ Telogen) and processed for IHC as described above [54].

### Wound healing assay

Mice were kept in standard housing conditions at the satellite animal facility. “Animal protocol was approved by Oregon State University Institutional Animal Care and Use Committee (IACUC), under permit number 3636”. For wound-healing studies, full-thickness excisional wounds, 5 mm in diameter, were generated on the dorsal side of 6–8 week-old adult female Ctip2^L2/L2^ and Ctip2^ep−/−^ mice (10 mice per genotype) using a 5 mm punch biopsy (Miltex Inc, YORK,USA). The wounds were imaged digitally each day and the diameter of each wound was measured using Photoshop (version 6.0; Adobe system, San Jose, CA). Healing was defined as the decrease in wound diameter over time and was expressed as the percentage of the day 0 wound diameter. Separate wound healing experiments were performed on Ctip2^L2/L2^ and Ctip2^ep−/−^ animals (8 animals of each genotype) per time and used for harvesting samples for protein extraction, histology and IHC. Briefly, the complete wound tissue with 2–3 mm border was excised around the wound at different time points (days 3, 5, 7, 9, 11 and 13) after injury, were bisected into two halves, one half immediately frozen for protein extraction and the other used for histology and IHC. Non wounded back skin was used as control. Most of the IHC were done on day 5 samples. Distance of the epithelial tongue migration from both sides was also measured. Data from each experiment were pooled. Immunoblot analysis on the skin extracts of wound healing samples was performed as described below. Statistical analysis was performed using the unpaired *t*-test and “two-way ANOVA” using the GraphPad Prism4 software.

### Whole skin extracts preparation and immunoblot analyses

Skin wound biopsies and samples from tape-stripping assays (at 0, 24 and 48 hours post tape stripping) were homogenized in Radio-Immunoprecipitation Assay [(RIPA buffer): 50 mM Tris, pH 7.5, 1% NP-40, 0.5% sodium deoxycholate, 0.1% SDS, 150 mM NaCl, 5 mM EDTA, proteinase inhibitors] using tissue grinder and cleared by centrifugation. Supernatants were collected and equal amounts of protein (25 µg, determined using the bicinchoninic acid (BCA:Pierce, Rockford, IL) protein assay) were subjected to SDS-PAGE, electro-blotted to nitrocellulose membranes, and were analyzed by immunoblotting using antibodies specific for the experiment. ß-actin was used as a loading control in these experiments. Membranes were blocked in 5% non-fat dry milk in Tris buffered saline (TBS) –Tween (T) (10 mM Tris–HCl, pH 7.8, 150 mM NaCl, 0.1% Tween 20), and incubated overnight with specific antibodies in the blocking buffer and developed using chemiluminescent substrate (GE Healthcare) using horseradish peroxidase-coupled secondary antibodies. The density of the band from the Western blots were quantified by using Multi Gauge v2.3 gel image analysis software (Fujifilm Corporation, Tokyo, Japan) and normalized by ß-actin.

### BrdU labeling and detection

Briefly, 6–8 weeks old adult mice were injected with 50 µg/g bodyweight of BrdU and skin biopsies were taken 2 hours after injection and processed as described [55]. 5 µm thick paraffin sections were stained with anti-BrdU (Serotech, Raleigh, NC, 1∶200) and anti-Ki67 (Abcam, Cambridge, MA,1∶500) antibodies to detect presence of proliferating S phase cells and those in G1, S, G2 phases of cell cycle, respectively.

### Histological analyses

For histological analysis the complete wounds including epithelial margins were isolated, bisected, fixed overnight in 4% paraformaldehyde in PBS, and embedded in paraffin. 5 µm thick paraffin sections were deparaffinized through graded series of xylene and ethanol, stained with either H &E or processed for IHC. Fontana–Masson staining was performed using a commercial kit according to the manufacturer's protocol (American MasterTech, Lodi, CA) as described [56].

Images were taken using Leica DME microscope and Leica DFC280 digital camera and analyzed using Leica Application suite v3.3.0 and Adobe Photoshop CS4.

### IHC analyses

IHC was performed on paraffin embedded sections as described previously [45], [46]. In brief, sections were deparaffinized in xylene, dehydrated through graded alcohols and a 20 minute, 750 W microwave pretreatment in citrate buffer (pH 6.0), and followed by treatment with 10% serum for 60 min. to block nonspecific antibody binding. The slides were then incubated with primary antibodies (anti-Ctip2, -K6, -K14, -K10, -Loricrin, -Filaggrin, -Ki-67, -PCNA, K15). Secondary antibody staining was carried out with a biotin-labeled antibody (Jackson Immuno Research Laboratories, Inc.) for 2 hrs. at 37°C, followed by incubation with a streptavidin-biotin horseradish peroxidase complex (Vector Laboratories, catalog number: SA-5704). Finally, sections were rinsed with phosphate buffered saline with tween (PBST), dehydrated through sequential washes in 50%, 70%, 95%, and 100% ethanol and then cleared in xylene. Slides were mounted with a mixture of Distyrene, a plasticizer, and xylene (DPX) and allowed to dry overnight. Images were taken as described in above section (histological analyses). Immunofluorescence studies utilized three washes with PBST after primary antibody incubation, and this was followed by incubation with fluorescently-labeled [Cy2 (1∶250) or Cy3 (1∶500) (Jackson ImmunoResearch)] secondary antibody for 2 hrs. Nuclei were counterstained with DAPI. Images were captured at 20× magnification using Leica DMRA fluorescent microscope and Hamamatsu C4742-95 digital camera and processed using OpenLab software and Adobe Photoshop CS4. IHC data was quantified using Adobe Photoshop CS4 and Image J software, and epidermal thickness was quantified using Leica One-Suite software. Multiple sections were analyzed from mice of each genotype and for each time-point, and significance was determined using a student's unpaired t-test.

### Antibodies

The following antibodies were used for IHC and/or western blot analysis: anti-Ctip2 (Abcam, 1∶300), anti-β-actin (Sigma, St. Louis, MO, 1∶3000), anti-K14 (Covance, Princeton, NJ, 1∶1000), anti-K10 (Covance, 1∶1000), anti-Ki67 (Abcam, 1∶500), anti-K6 (Abcam, 1∶ 200), anti K15 (Covance, 1∶1000) anti-Loricrin (Covance, 1∶1000), anti-Filaggrin (Covance, 1∶1000), anti-CK16 (Abcam, 1∶2500), anti K8 (Troma-1; Developmental Studies Hybridoma Bank, Ames; 1∶200), anti-PCNA (Abcam, 1∶1500),anti-BrdU (Serotech, Raleigh, NC, 1∶200), NFATc1 (Santa Cruz Biotechnology, Inc., CA, 1∶200), CD34 (Abcam, 1∶200), LRIG1(R&D Systems, Minneapolis, MN, 1∶500), CD133 (Abcam, 1∶200), E-cadherin (cell signaling, Danvers, MA, 1∶200), Phalloidin (Sigma, 1∶1000) and alpha SMA (Abcam 1∶100).

### Statistical analyses

Statistical significance of differences between different groups was assessed using two-tailed unpaired t-test and GraphPad Prism software. A two-way ANOVA analysis was performed for excisional wound closure data, and Student's *t*-test was utilized to analyze the data for migratory tongue distances, epidermal thickness and for quantification of Ctip2, BrdU, Ki-67 and PCNA positive cells in skin. Quantification of Ctip2, Ki67- or PCNA-positive cells of control and mutant mice was determined by counting total number of Ki67/PCNA-positive cells, and expressed as a percentage of DAPI^+^ cells. Quantification of alpha SMA-positive cells in was done using ImageJ (NIH, MD, USA). The analysis was by estimation of signal intensity of SMA-positive cells selectively in the granulation region of dermis of the skin samples, excluding blood vessels. Data obtained from each group of control and mutant mice for each time point were combined for calculating the mean data and SEM. All statistical analyses were independently performed by blinded investigators.

## Results

### Ctip2 expression is induced in adult mouse skin epidermis in response to mechanical injury and wounding

**Figure 1:**
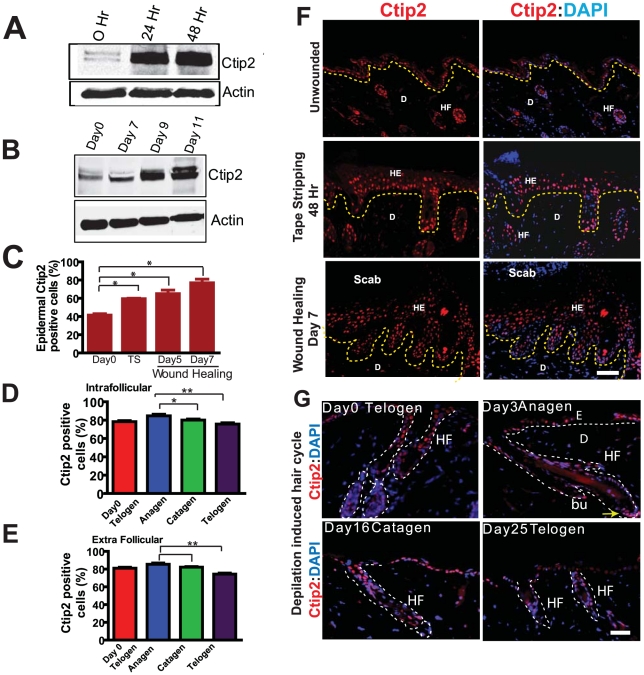
Expression of Ctip2 in skin of adult mice following tape stripping (TS), during full thickness wound healing and in hair cycling. Immunoblot analyses of Ctip2 protein expression in wild type mice skin 24 and 48 hrs post tape stripping (**A**), and in wound biopsies obtained at Days 7, 9 and 11 after wounding (**B**). β-actin is used as control. (**C**) Bar chart showing an increase in the percentage of Ctip2 positive cells 48 hours post TS and on days 5 & 7 post wounding (*p<0.05). Bar chart showing, percentage of (**D**) intrafollicular and (**E**) extrafollicular Ctip2 expressing cells in depilation induced hair cycling in adult mouse skin. Significant (* P<0.05; **P<0.005) increase in Ctip2 expression was observed in induced anagen compared to telogen and catagen stages (D and E). (**F**) An overview of Ctip2 localization by immunofluorescence (IF) in the adult unwounded mice skin, 48 hr after mechanical injury and at day 7 post wounding using anti-Ctip2 antibody. (**G**) Overview of Ctip2 localization by IF in depilation induced hair cycle in mice skin. HE-hyperproliferative epithelium; HF-hair follicle; E-epidermis; D-dermis; Bu-bulge; DP-dermal papilla; bu-bulge. Yellow dotted lines separates the epidermis from dermis (F) and white dotted lines outlines the HF (G). Scale bars: 50 µm (F) and 25 µm (G).

Ctip2 is highly expressed in mouse skin during embryogenesis and in adult human skin [42], [45]. Adult mice skin contains low levels of Ctip2 compared to embryonic or neonatal skin [42]. CTIP2 has been reported to be overexpressed in human head and neck cancer and its expression is linked to poorly differentiated tumor status [46]. Above results and the link between chronic wounds and cancer, led us to hypothesize that Ctip2 has important function(s) during adult tissue injury and in wound healing. Mechanical injury by tape stripping induces transient epidermal hyperplasia followed by changes in proliferation and differentiation status of the epidermis [44], [57]. To this end, we performed tape stripping and wound healing in wild type adult mice [18]. As expected, Ctip2 was expressed in the epidermal keratinocytes in unwounded skin at very low levels (Fig. 1A). Ctip2 expression was observed to increase after 24 and 48 hours post tape stripping in wild type mice skin by immunoblotting, and on wounding at 7, 9 and 11 days post wounding compared to unwounded skin (Fig. 1A, B and C). Analyses of Ctip2 expression by IHC in post-tape stripped and wound-healing skin biopsies revealed most intense Ctip2 expression in hyper proliferative epidermis (HE) (Fig. 1F, see 48 hrs. post tape stripped and Day 7 wound healing epithelium) compared to normal epidermis. Both mitotically dividing epidermal basal cells as well as the post-mitotic suprabasal cells expressed high levels of Ctip2 during and in later phases of cutaneous healing (Fig. 1F). Percent Ctip2-positive cells was increased in the epidermis of both tape stripped skin (59.5±0.5%) and in full-thickness wounds (day 5: 65±4%; day 7: 77±4%) compared to the unwounded skin (41.5±1.5%; see Fig. 1C). In order to evaluate if Ctip2 expression is linked to hair cycle stages, we determined Ctip2 expression in both depilation induced hair cycle and normal hair cycle in dorsal skin of wild type mice (Fig. 1D, G, E and Fig. S1 C and D). IHC analysis of depilation induced hair cycle revealed that mice anagen HFs had predominant expression of Ctip2 in the proliferative hair germ (indicated by yellow arrows in Fig. 1G) and epithelia in outer root sheath (ORS) of the HF (Fig. 1G). Ctip2 expression was significantly reduced in catagen and telogen phase (Fig. 1E and G). Percentage of intrafollicular Ctip2 expression was highest in the depilation induced anagen followed by catagen and telogen (Mean ± SEM of anagen, catagen and telogen are: 84.69±1.8; 80.15±1.0; 74.21±1.923) (Fig. 1D). IHC staining of Ctip2 in normal hair cycle stages 2^nd^ anagen (P28) and 2^nd^ telogen (P49) did not reveal significant differences in Ctip2 expression in the intra- and extra-follicular compartments of the skin (Fig. S1, C and D) [54]. Ctip2 expression was also analyzed in RA and TPA induced proliferative skin. We observed an increased epidermal thickness with a concomitant increase in Ki67- positive proliferating keratinocytes in basal and suprabasal layers but not in Ctip2 expression, suggesting that Ctip2 is linked with cell proliferation, but is not induced post TPA or RA treatment (Fig. S1A and B). Overall, these results confirm that Ctip2 expression is induced post tape stripping, during wound healing and in anagen phase of induced hair cycle, suggesting a role of Ctip2 in these processes.

### Selective deletion of Ctip2 in epidermal keratinocytes delays cutaneous wound healing

Epidermal keratinocytes are key players in wound closure and are required for the re-epithelialization step. *In vitro* scratch assays mimic the *in vivo* wound re-epithelialization process and commonly used to measure the motility of adherent cells [48]. We therefore performed scratch migration assay on isolated primary keratinocytes from wild type and Ctip2 null neonatal mice skin to determine function(s) of keratinocytic Ctip2 in wound closure. Assay was performed in presence of mitomycin-C (MMC) to block cell proliferation, which enabled further evaluation of Ctip2 effects on cell migration, while excluding any influence of cell proliferation [58], [59]. Overall, migration of Ctip2 null keratinocytes was reduced in comparison to control cells and the difference became statistically significant (p<0.05) at day 3 (Fig. S2A). MMC at 1 µg/ml and 5 µg/ml could inhibit cell proliferation. Both concentration of MMC equally reduced keratinocyte migration (days 3 and 4) significantly in wild type and mutant keratinocytes compared to untreated cells (Fig. S2A). Similar results were obtained by *in vitro* transwell migration assays (Fig. S2B). Results from both assays suggested that Ctip2 was necessary for efficient wound closure.

Since Ctip2-null mice die within 6–8 hours after birth, we generated Ctip2^ep−/−^ mice, in which Ctip2 was selectively deleted in the epidermis using Cre-loxP strategy and the K14-Cre mice, for further *in vivo* analyses [60], [61]. The Ctip2^ep−/−^ young adult mice skin had Ctip2 deletion in most of the areas except some residual staining, which was occasionally detected by IHC but not by immunoblotting, indicating that constitutively expressed Cre recombinase induced a strong deletion of Ctip2 in most cells of the epidermis (Fig. S3 A and B). Proliferation assay using BrdU labeling revealed increased number of BrdU positive cells in Ctip2^ep−/−^ epidermis (4.993±1.1) compared to the control epidermis (2.481±0.8) (Fig. S3 C and D). Similar trend was observed for other proliferation marker Ki-67 (Ctip2^L2/L2^ 9.628±0.9 and Ctip2^ep−/−^11.23±1.5) by IHC, although the increase was not statistically significant (P>0.05) (Fig. S3 D). Histological analyses of dorsal skin biopsies from 6–8 week-old Ctip2^L2/L2^ (control) and Ctip2^ep−/−^ (mutant) mice revealed a significantly increased epidermal thickness in mutants (mutant: 21.0±0.9 µm; control: 15.0±1.0 µm) compared to control mice (Fig. S3E). Epidermal staining for keratinocyte basal cell marker K14 was more intense in mutants compared to control skin (Fig. S3F). Furthermore, immunoblot analyses confirmed that mutant skin exhibited an overall higher expression of proliferation marker PCNA (Fig. S4A). Although, no significant difference was observed for expression of early differentiation marker K10 in mutant epidermis, the late differentiating marker filaggrin and loricrin were more uniformly expressed in Ctip2^ep−/−^ mice (data not shown). Altogether, these results suggest that lack of Ctip2 in young adult epidermis alters epidermal homeostasis in unwounded skin.

**Figure 2:**
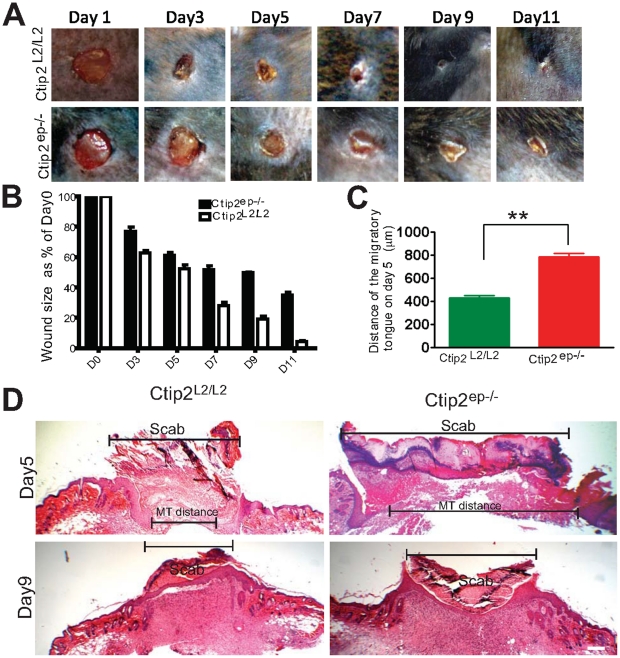
Impaired wound healing in skin of adult Ctip2^ep−/−^ mice. (**A**) Macroscopic images of time course of healing of 5 mm full-thickness excisional wounds. Note, that in Ctip2^L2/L2^ mice at day 7, 9 and 11 post-wounding, the wound area was significantly reduced compared to the Ctip2^ep−/−^ mouse. (**B**) At indicated time points, the diameter of the wound was measured using photography and digital analyses. Data from three independent experiments (9 Ctip2^L2/L2^ mice and 9 Ctip2^ep−/−^ mice were used for this study) were pooled. The difference in the rate of wound closure was statistically significant between two groups (P<0.001). (**C**) Graph showing increased migratory tongue distances between the two migratory epithelial tongue on the wound bed from Ctip2^L2/L2^ and Ctip2^ep−/−^ mice. (*p<0.05). (**D**) Hematoxylin and Eosin (H & E) stained images of the day 5 and 9 post wounding samples. The black broken brackets in day 5 and day 9 show the outline of the wound area and the migratory tongue distance (day 5). Hyperproliferative epithelium; E- Epidermis; D-Dermis. Scale bar: 200 µm (**D**).

We hypothesized that keratinocytic Ctip2 modulate cutaneous wound healing in a cell-autonomous manner. Therefore, we studied *in vivo* cutaneous wound healing processes in Ctip2^ep−/−^ mice as described in materials and methods section. In the post wounding days, mutant mice demonstrated significantly more open areas compared to the wild type counterparts (Fig. 2A and B). Quantitative analysis of wound diameter revealed significantly delayed wound healing in Ctip2^ep−/−^ mutant mice compared to Ctip2^L2/L2^controls (p<0.001; Fig. 2B). Re-epithelialization efficiency of the wounds was determined by comparing the gaps between the migratory tongue from both sides in control and the Ctip2^ep−/−^ mutant skin [20], [62], [63]. Wound re-epithelialization was delayed in mutants compared to the Ctip2^L2/L2^ control mice as judged by the distance between the migratory tongue (Ctip2^ep−/−^: Mean ± SEM 787.8±26; Ctip2^L2/L2^: Mean ± SEM 431.3±20) (Fig. 2C and D). A thin migratory tongue was visible on both sides of the Ctip2^L2/L2^ control wound margin at day 5, whereas the mutant epidermis had become thicker and blunted (Fig. 2D). Increased melanocyte proliferation has been reported in the regenerating wound epidermis of neonatal mice skin until day 7 post wounding [64], [65]. No differences in pigmentary activity were observed between the control and mutant skin pre- and post wounding (Days 5 and 7) by Fontana Masson staining [56], [66]; data not shown). Altogether, these results indicate that loss of Ctip2 in the epidermis delays rate of re-epithelialization and establish a cell autonomous role of Ctip2 in keratinocytes during would healing and skin regeneration.

### Impaired keratinocyte activation, altered cell proliferation and delayed onset of differentiation in Ctip2^ep−/−^ skin during wound healing

**Figure 3:**
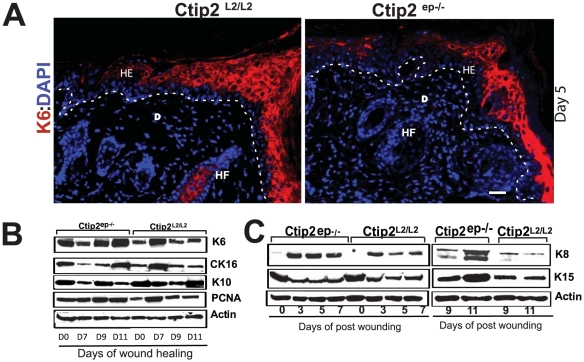
Altered keratinocyte activation (K6 & K16), proliferation (PCNA), differentiation (K10 & K8), and impaired expression of epithelial stem cell marker (K15) during wound healing in mutant mice. (**A**) IHC analysis of K6 (red) expression on Day 5 post wounding samples from Ctip2^L2/L2^ and Ctip2^ep−/−^ mice. The white dotted lines indicate the migratory tongue on the wound bed (Day5 samples) and separation of the epidermis from dermis in (A). All sections were counterstained with DAPI (blue). HE- hyperproliferative epithelium; E- epidermis; D- dermis; HF- hair follicle. Scale bar: 50 µm. (**B**) Immunoblot analyses of PCNA, K10, K6 and K16 in adult skin of Ctip2^L2/L2^ and Ctip2^ep−/−^ mice after wounding (Day 7, Day 9 and Day 11). (**C**) Immunoblot analyses of keratin markers, K8 and K15 induction in the skin of Ctip2^L2/L2^ and Ctip2^ep−/−^ adult mice on days 3, 5, 7, 9 & 11 post wounding. * indicates undetectable K8 expression in the unwounded wild type skin (day 0). β-actin is used as an internal control.

Upon injury, keratinocytes in suprabasal compartment nearest to the wound edge become activated, resulting in morphological changes and induction of marker gene expression [67], [68], [69]. Activated keratinocyte markers K6 and K16 that are not expressed in normal epidermis are expressed in migrating keratinocytes during wound healing and Keratin 8 (K8) is expressed in simple epithelia and not in intact epidermis or at wound margins. Day 5 wounds revealed uniform K6 staining throughout the newly formed migratory tongue and newly formed epidermis in control Ctip2^L2/L2^ but not in Ctip2^ep−/−^ mutant mice (see Fig. 3A, day5). Interestingly, by immunoblot analyses day 7 wounds showed an induction in K6 and K16 expression in Ctip2^L2/L2^ controls but not in mutant wounds, and that induction was delayed until day 9 and day 11 in mutant wounds (Fig. 3B). Although, unwounded mutant skin exhibited an overall higher expression of K6 and K16 compared to the control skin (Fig. 3B and Fig. S4A). As reported earlier, K8 was not detected in the control skin but was clearly detectable in Ctip2^ep−/−^ unwounded skin (Fig. 3C). In Ctip2^L2/L2^ controls wounds, induction of K8 was detected at days 3–7 post wounding (Fig. 3C, no expression in day 0, indicated by *). Whereas in mutant wounds, K8 induction was much higher and remained elevated in later days post wounding (Fig. 3C; see day 7). Our present results suggested that mutant skin exhibit higher and sustained induction of K8 and delayed keratinocyte activation post wounding.

**Figure 4:**
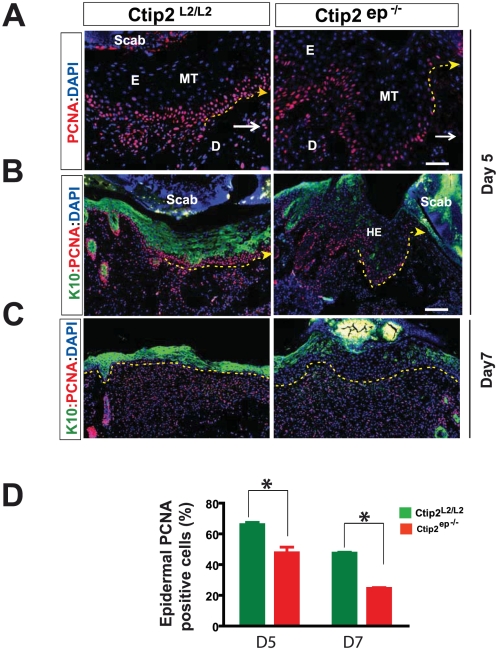
Reduced epidermal proliferation and differentiation in skin wounds from Ctip2^ep−/−^ mice. (**A–C**) Expression of PCNA in skin sections from Ctip2^L2/L2^ and Ctip2^ep−/−^ mice on day 5 (A and B) and day 7(C) post wounding, using antibodies against PCNA (in red). (**B** &**C**) Co-localization of K10 and PCNA on days 5 and 7 wound biopsies from dorsal skin of Ctip2^L2/L2^ and Ctip2^ep−/−^ mice using anti-K10 antibody (green) and PCNA (red). All slides were counterstained with DAPI. The white arrow points to the directions of the wound (A). Yellow arrow with dotted lines indicates the direction of wound (B) and separates the epidermis from the dermis (C). MT-migratory tongue; HE- hyperproliferative epithelium; HF- hair follicle; E-epidermis and D- dermis. (**D**) Bar graph showing significant decrease (*P<0.05) in percentage of PCNA positive cells during wound healing on day 5 and 7 in the skin of Ctip2^ep−/−^ mice. Green and red bars represent Ctip2^L2/L2^ and Ctip2^ep−/−^ mice, respectively. Scale bars: 50 µm (A), 100 µm (B and C).

It is possible that the delay in wound closure in Ctip2^ep−/−^ mice is also due to differences in proliferation at the wound edge or in the migrating tip of both wounds. Indeed, IHC analyses for PCNA revealed a significant decrease in the number of proliferating keratinocytes in mutant epidermis at days 5 and 7 post-wounding compared to Ctip2^L2/L2^ controls skin (Fig. 4A–D). Western blotting also revealed a modest difference in induction of proliferation marker PCNA between Ctip2^L2/L2^ controls and mutant skin wounds on post-injury day 7 (Fig. 3B). However, PCNA expression in Ctip2^ep−/−^ skin remained elevated at later days (day 11) of healing unlike in wild type where its expression was reduced to the basal level (Fig. 4A). These results suggest that loss of keratinocytic Ctip2 resulted in a delayed but persistent induction of proliferative cells in the epithelial tongues during wound closure.

K10, a marker for cell differentiation, is expressed in all suprabasal keratinocytes in a graded pattern. K10 expression was detected in the migratory tongue adjacent to the wound at day 5 in the Ctip2^L2/L2^ controls mice. However, K10 expression was greatly reduced in the delayed migratory tongue and epidermis adjacent to the wound of Ctip2^ep−/−^mutant mice at all days post-wounding (Fig. 4B & C). By day 7 post-injury, expression of K10 in newly formed epidermis of control skin was uniform in all the suprabasal layers (Fig. 4C, left panel), whereas its expression was discontinuous and not normalized in the neo-epidermis of mutant mice (Fig. 4C, right panel). Altogether, these results confirm that Ctip2 mutant skin had reduced proliferation at the migratory tongue and reduced differentiation of the neo-epidermis. In addition, it is conceivable that sustained keratinocyte proliferation observed in Ctip2^ep−/−^ epidermis impedes differentiation within the suprabasal epidermal layer of these mice. Overall, results suggest an important role of Ctip2 in regulating keratinocyte activation, proliferation and differentiation during wound repair.

### Lack of E-cadherin suppression in the migratory tongue and insufficient expression of alpha SMA in the dermis of Ctip2^ep−/−^ mice skin during wound repair

**Figure 5:**
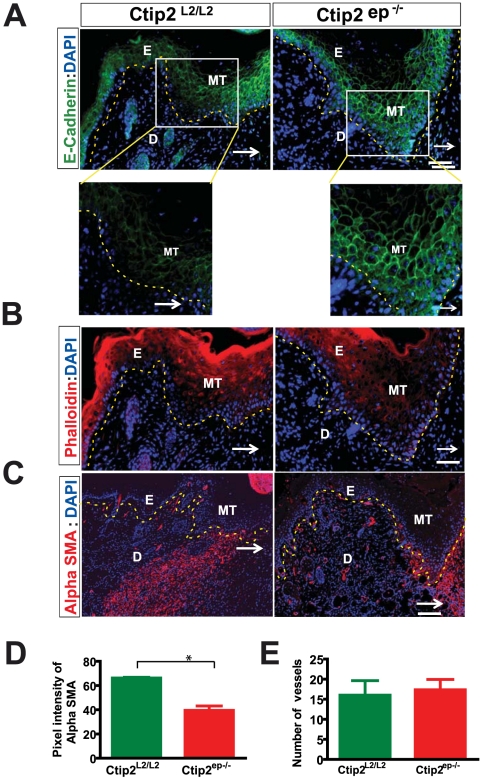
Altered expression of E-cadherin, Phalloidin and alpha SMA in Ctip2^ep−/−^ mutant day 5 skin wounds. (**A**) Expression of E-Cadherin is down regulated in the Ctip2^L2/L2^ control basal layer migratory tongue but not in Ctip2^ep−/−^ mutants. The insets are magnified. (**B**) Expression of Phalloidin (red) is down regulated in the Ctip2^L2/L2^ control but not in Ctip2^ep−/−^ mutant migratory tongue. (**C**) Reduced accumulation of myofibroblast (alpha SMA: red) in Ctip2^ep−/−^ mice wounds compared to the wild type mice. (**D**) Quantification of alpha SMA. Bar graph shows reduced pixel intensities in Ctip2^ep−/−^ compared to the Ctip2^L2/L2^ control. Data are represented as means ± SD. Significant differences between Ctip2^L2/L2^ and Ctip2^ep−/−^ (*P*<0.05). (**E**) Increased numbers of blood vessels in Day 5 post wounding samples inCtip2^L2/L2^ compared to Ctip2^ep−/−^ mice but not significant. Values are the mean and SEM (n = 4 mice). MT-migratory tongue; E-epidermis and D- dermis. Arrows point to the directions of the wound. Dotted lines demarcate the epidermis from the dermis. Scale bars: 50 µm (A and B), 100 µm (C).

Alterations in cell–cell adhesion is a known phenomenon during re-epithelialization stage of wound healing, and granulation tissue formation is important for the remodeling phase [11]. Since Ctip2^ep−/−^ mice wounds displayed delay in migration, we examined expression of E-cadherin as a marker of adherens junctions and F-actin distribution during wound healing process at day 5 post wounding. The migratory epithelium of Day 5 Ctip2^L2/L2^ skin wounds exhibited loss of E-cadherin staining in the cell-cell borders of basal cells compared to the Ctip2^ep−/−^ mutant wounds, where E-cadherins were readily detectable at cell-cell borders and expression appeared to be stronger (Fig. 5A). In contrast, F-actin distribution (visualization of actin microfilaments) appeared to be normal and well organized actin cytoskeleton was similar in both control and mutant skin as demonstrated by similar expression pattern of Phalloidin in both genotypes (Fig. 5B). Since alpha SMA expressing myofibroblast are responsible for development of extra cellular matrix (ECM) post inflammation during wound healing, we looked at its expression on day 5 wounds. Interestingly, the day 5 Ctip2^L2/L2^ control skin wounds had stronger alpha SMA expression in the dermis in the granulation tissue (Ctip2^L2/L2^ controls: Mean ± SEM 66.24±0.6015) (See Fig. 5 C and D). In contrast, Ctip2^ep−/−^mutant wounds expressed less alpha SMA in the granulation tissue (Ctip2^ep−/−^ mutants: Mean ± SEM 39.48±3.687). Although the alpha SMA positive vessel numbers were slightly higher in the Ctip2^ep−/−^ mutants (Mean± SEM 17.33±2.603) compared to Ctip2^L2/L2^ controls (Mean± SEM 16.00±3.606), it was not statistically significant. Altogether, results suggest altered cell-cell adhesion and impaired ECM development in healing wounds of Ctip2^ep−/−^ mutant mice.

### Altered expression of epithelial stem cell markers during wound healing in mutant mice

**Figure 6:**
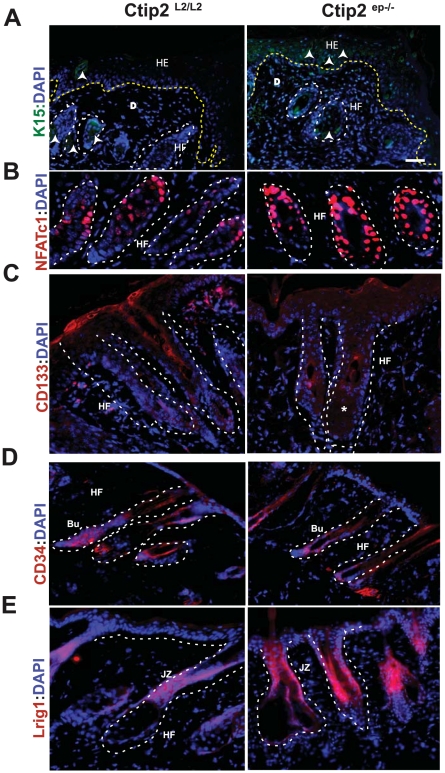
Aberrant expression of markers of HF stem cells in Ctip2^ep−/−^ mice skin during wound repair. IHC analysis of (**A**) K15, (**B**) NFATc1 (red), (**C**) CD133 and (**D**) CD34 and (**E**) Lirg1 expression in HF on day 5 post wound healing samples from Ctip2^L2/L2^ and Ctip2^ep−/−^ mice, using specific antibodies (* indicates loss of CD133 positive cells). The yellow dotted lines indicate the separation of epidermis from dermis near the wound bed (Day5 samples) in (A) and white dotted lines outline the HF. All sections were counterstained with DAPI (blue). Arrow heads indicate K15 positive cells (A). HE-hyperproliferative epithelium; E- epidermis; D- dermis; HF- Hair follicle; Bu-Bulge; JZ-junctional zone. Scale bars: 50 µm.

Adult stem cells (ASCs) of the skin are source for lost cells during wound healing and thus are the key players in tissue regeneration. We therefore looked at the effects of Ctip2 deletion on expression of various cutaneous stem cell markers such as K15, NAFTc1, CD34, CD133, Lgr5, and Lrig1 in healing wounds at day 5 post wounding. In the control wound adjacent epidermis, K15 level was down regulated as expected, but the mutant wound adjacent epidermis had patchy expressions of K15 (Fig. 6A). Immunoblot analyses exhibited an overall reduction in K15 levels after wounding in both Ctip2^L2/L2^ control and Ctip2^ep−/−^ mutant epidermis (Fig. 3C; days 3–7). However, K15 expression was restored to basal levels earlier (day 11) in wound-adjacent epidermis of Ctip2^ep−/−^ mice, compared to control mice (see Fig. 3C). Interestingly, all Day 5 wounds had elevated expression of NFATc1 in the hair HFs of mutant mice compared to control mice (Fig. 6B). Western blot analyses confirmed that total NFATc1 protein was upregulated post wounding, as multiple bands were visible due to the presence of different isoforms (Fig. S3F, Fig. S4 B). Similarly, CD133 expression was induced in the wound adjacent hair HFs of Ctip2^L2/L2^ control mice but its expression was lacking in wound adjacent mutant HF at day 5 (Fig. 6C). Western blot analyses also confirmed induction of CD133 at day 5 in control skin, but its induction was delayed until day13 in mutant skin (Fig. S3F and Fig. S4B). Expression of another stem cell marker CD34 was modestly reduced in the bulge region of the mutant HF compared to the control follicles (Fig. 6D). Immunoblot analyses showed reduced CD34 induction at day 5 in Ctip2^ep−/−^ mice, and the levels were not normalized up to day 13 (Fig. S3F and Fig. S4B). Interestingly, robust induction of Lrig1 expression was observed in the junctional zone of the mutant HF compared to control follicles at day 5 and that increase was maintained until day 13 post wounding (Fig. 6E and Fig. S3F and Fig. S4B). Altogether, our results confirm aberrant expression pattern of a subset of cutaneous stem cells markers in mutant skin during wound healing.

## Discussion

To our knowledge, the role of Ctip2 in regulating the steps of injury abrasion and wound healing has not been previously reported. Ctip2 is linked with cell proliferation, its expression increases proportionately with increase in cell number but is not induced post TPA or RA treatment. Ctip2 levels also changes with induced hair cycle, with highest increase in anagen phase and lowest in catagen phase, possibly regulating hair cycling. Ctip2 is induced in transient epidermal hyperplasia induced by mechanical injury, suggesting its importance in adult mice skin when cells undergo rapid turnover. Similar CTIP2 upregulation has been reported in epidermis of hyperproliferative diseases such as atopic dermatitis (AD) and allergic contact dermatitis (ACD) [45], supporting our assumption that Ctip2 function is implicated in epidermal regeneration and re-establishment of skin homeostasis under pathological conditions. We expected to see epidermal hypoplasia in absence of Ctip2 in Ctip2^ep−/−^ mice, instead, we observed a compensatory hyperplasia in unwounded skin of these mice, which could be attributed to impaired development of protective epidermal barrier, and enhanced trans-epidermal water loss (TEWL) [our unpublished data; [44]]. The effects of Ctip2 deletion was analyzed in in-vivo full-thickness wound-healing processes and the results suggest defects in proliferation, migration, cell-cell contacts and proper expression of HF stem cell markers.

The delayed wound re-epithelialization and blunted migratory tongue observed during wound healing in Ctip2^ep−/−^ mice can be explained in a number of ways. The hyperthickened epidermis at the wound-edge in the mutants at day 5 indicated keratinocyte proliferation without sufficient migration. We observed lower percentages of PCNA positive keratinocytes on days 5 and 7 post-wounding in the mutants during the early phases of wound healing process and sustained proliferation in the later days of healing. In spite of the observed delay in re-epithelialization, eventually these keratinocytes finally migrated over the wound bed to complete re-epithelialization, suggesting that mutant keratinocytes must be lacking factor(s), such as heparin binding epidermal growth factor (HbEGF), which can stimulate keratinocyte migration during wound healing [70], [71]. We reported earlier that skin of Ctip2 null mice expressed less HbEGF than control mice skin, thus, decreased motility of Ctip2–null keratinocytes may be partly due to defective autocrine/paracrine EGF signaling pathway [44]. It seems likely that other factors eventually compensate for lack of Ctip2, and complete the migration process. The ability of Ctip2 to regulate other signaling pathways, such as that involving AP-1 [44], could also be responsible for defects in wound closure [70], [71]. We have observed Ctip1 induction in the mutant mice post injury which might be compensating for loss of Ctip2 and helping the re-epithelialization process later during healing (data not shown). In order to be able to migrate, keratinocytes undergo a preparative phase for re-epithelialization, which is accompanied by alteration of the cell-cell adhesion mediated by E- and/or P-cadherin [11]. Down regulation of E-cadherin expression has been associated to migration of cancer cells and during wound re-epithelialization process [10], [11]. E-cadherin is downregulated at the basal layer of epidermis in the wound margin keratinocytes during wound repair processes [10], [11]. In agreement with previous report, we observed it's down regulation in the wound margin keratinocytes in control mice but not in the mutant wounds. Our results suggest that cell-cell contact appear tighter in the mutants when compared to loosely packed cells in the control wounds, thereby inhibiting keratinocyte migration and re-epithelialization. Similar observations were also reported in β1-integrin null mice having keratinocytes with impaired migration and tightly packed hyperproliferative epithelium [72]. Interestingly, E-Cadherin suppression has been reported to direct cytoskeletal rearrangement and intraepithelial tumor cell migration in 3D human skin equivalents [10].

Normal differentiation after wound healing is marked by restoration of expression of differentiation markers (K10, loricrin and involucrin) in the neo-epidermis. Reduced expression of K10 in the mutant neo-epidermis suggested a delay in restoration of the normal differentiation program. K10 expression is lowest at the wound edge and increasingly higher levels of K10 are expressed radiating out from the wound edge [67], [73]. Expression of differentiation markers K10 and filaggrin was also strongly reduced in Ctip2^−/−^ neonatal mice, which affected the differentiation program [44]. Wounds in Ctip2^ep−/−^ mutants exhibited a delayed K6 induction and a delay in K15 suppression, leading to an overall delay in activation, and hence a slower rate of cell migration and re-epithelialization. During wound healing, K6, K16 and K15 are differentially expressed. K15 expression is repressed during the first few days of wound healing whereas K6 and K16 expression increases in the same time window [74]. One of the important features of hyperproliferative healing epidermis is the induction of K6 during wound healing [3], [32], [72]. In the present study, K6 and K16 induction occurs much later in the wound-healing process in Ctip2^ep−/−^ mutants compared to the control mice. Reduced K6 expression has been linked to delay in wound healing in transgenic mice overexpressing stem cell marker Necl2 [32]. Patches of K15 expression were also detected in the wound bed at day 5 post wounding in mutants, suggesting that these keratinocytes were less activated (confirmed by low K6 expression in the same cells). Such premature expression of K15 indicates a degree of early maturation of basal cells [22]. It is possible that although the mutant epidermis proliferated appropriately, the timing of expression of keratin markers K6 and K15 in mutant keratinocytes was out of phase resulting in a delay in wound closure [18], [74]. Suppression of K15 expression by growth factors and cytokines has been reported from both *in vitro* and *in vivo* cutaneous injury studies [74].

Interestingly, robust induction of K8 expression in Ctip2^ep−/−^ mutant epidermis is indicative of faulty K8 regulation, and disruption of epidermal differentiation program. Elevated K8 expression is associated with increased migration and invasive capacities in some situations [75], . Sporadic expression of K8 in HF has been reported earlier and could explain the induction of this gene by wounding in the wild type mouse skin [21]. Alteration in morphology and differentiation of epidermis and HFs was seen in mice over-expressing human K8 in the skin [21]. Increased expression of K8 in Slug mutant mice lead to compromised re-epithelialization similar to Ctip2 mutant mice, suggesting that elevated K8 may not support re-epithelialization [7]. Interestingly, Slug level was unaltered in skin of Ctip2 mutant mice and Ctip2 was not recruited on K8 promoter region suggesting a yet unidentified mechanism(s) of its regulation by Ctip2 (data not shown).

Furthermore, myofibroblasts are required in normal wound healing for wound contraction and ECM formation. Ctip2 is highly expressed in dermal fibroblasts during skin organogenesis and Ctip2 expression is induced in the dermis during cutaneous wound healing, suggesting that this transcription factor might play a role in dermal homeostasis or response to wounding. Presence of αSMA-positive myofibroblasts both in the Ctip2^L2/L2^ and Ctip2^ep−/−^ mutants wounds corroborates well with previously published results [78], [79]. However, reduction of myofibroblast differentiation (confirmed by low expression of alpha SMA) in the granulation tissue of healing wounds from Ctip2^ep−/−^ mutant mice at day 5 post wounding suggests that, although the myofibroblast cells are recruited in the healing process they are not sufficient enough for normal healing. Our results indicate a non-cell autonomous role of keratinocytic Ctip2 in mediating myofibroblast differentiation. Therefore, actual contribution of Ctip2 in dermal fibroblasts during wound healing might need to be delineated following selective ablation of Ctip2 in this cell compartment using specific Cre-deletor strains.

Most importantly, we have seen that several cutaneous stem cell markers (K15, NFATc1, CD34 and CD133) were aberrantly expressed during wound healing in Ctip2^ep−/−^ mice. Keratinocytes that re-epithelialize the wound are derived from interfollicular epithelial stem cells (epiSC) and from the hair bulge, and Ctip2 is expressed in both compartments. We have previously shown that Ctip2 co-localizes with most of the CD34 expressing cells in HF. In this study, we have shown that post wounding, day 5 epidermis in Ctip2^ep−/−^ mice (lacking Ctip2 in epidermis) has low levels of CD34 in wound adjacent HF suggesting lack of sufficient availability of CD34 positive cells for the normal healing of the mutant skin [42]. It has been shown that co-transplantation of CD34(+) cells with CD34(+) endothelial cells improved wound healing [80]. Therefore, absence of CD34+ cell population in mutants could be one of the factors for delayed healing. In an improved method of human keratinocyte culture from skin explants, cells that migrated out from explants strongly expressed markers K15 and CD133 and displayed intense K6 expression, indicative of activated keratinocytes in wound-healing epidermis [39]. Therefore, lack of CD133 positive cells in the Ctip2 mutant skin HF can contribute, at least in part, to delayed re-epithelialization and wound closure. Stem cell marker NFATc1 is expressed by quiescent adult stem cells of the HF. Suppression of NFATc1 signaling pharmacologically or by conditional *NFATc1* gene ablation, activated stem cells prematurely, resulting in premature follicular growth [33]. In our study, parallel NFATc1 induction was noted in mutant mice at post wounding day 5, suggesting less activation of stem cell population available to re-populate the wound bed. Similarly, Lrig1 overexpression has been shown to have growth inhibitory effects by inhibiting ERK activation and cell cycle progression. During development as well as in postnatal stages, epidermal growth factor receptor (EGFR) signaling controls important cellular programs such as survival, proliferation and differentiation. Lrig1 is one of the inducible feedback inhibitors (IFIs) that bind to EGFR and suppress receptor signaling through several mechanisms [81], [82]. We observed modest increase in Lrig1 expression in the unwounded skin of mutant mice, and these mice had robust induction of Lrig1 post wounding day 5. Interestingly, Ctip2 null keratincytes express low levels of EGFR (Zhang et al., unpublished data). EGFR has been implicated in the regulating multiple facets of wound healing including proliferation, migration and wound contraction [83]. Therefore, loss of EGFR can explain, at least in part, the delay observed in cutaneous wound healing in Ctip2**^ep−/−^** mice. Altogether, altered expression of many of the HF stem markers were observed in these mice and leaves a potential scope for future studies on how Ctip2 regulates stem cell mobilization during post wounding by deleting Ctip2 in the specific stem cell compartment(s) using individual stem cell lineage specific Cre-driver mice.

In conclusion, our current studies suggest that Ctip2 in keratinocytes might be an important player in wound repair processes in otherwise healthy patients, as well as those with chronic diseases, such as diabetes. A more complete understanding of contribution of Ctip2 mediated regulatory controls of cutaneous stem cells, inflammation and hair cycling during wound healing is necessary for better control of wound repair and tissue regeneration. Understanding the mechanisms of Ctip2 mediated regulation of tissue remodeling is likely to provide novel treatment strategies for improved wound healing.

## Supporting Information

Figure S1 **Ctip2 expression in TPA & RA induced skin hyperproliferation and in different stages of hair cycling.** (**A**) Immunoflourescence staining of Ctip2 (red) and Ki-67 (green) in TPA and RA treated skin of the wild type mice. All slides were counterstained with DAPI (blue). The yellow dotted lines indicate separation of the epidermis from dermis. (**B**) Quantification of epidermal Ctip2 and Ki-67 in TPA and RA treated wild type skin. # indicates that the Ctip2 percentages are not significant between the treatment groups. * indicates that the Ki-67 percentages are significant between the groups (p<005).(**C**) Immunoflourescence staining of Ctip2 (red) in skin of wild type mice during normal hair cycle stages 2^nd^ anagen and 2^nd^ Telogen. (**D**) Quantification of extrafollicular and intrafollicular Ctip2+ cells during natural hair cycle stages do not show significant difference in their expression pattern. Scale bars: 50 µm (A), 100 µm (C).
(EPS)

Figure S2 **Reduced migration of Ctip2^−/−^ keratinocytes in** ***in vitro*** **scratch migration and transwell migration assay.** Scratch migration assay in keratinocytes were performed in presence of 1 µg/ml and 5 µg/ml of MMC. After scratches were introduced, cell migration and wound closure were monitored at indicated times (Day 0, 1, 2, 3 and 4). For transwell assay, keratinocytes were seeded at a density of 3×10^5^ cells/cm^2^ into the upper wells of transwell inserts. After one or two days cells in the upper wells were removed by cotton tip swabs, and cells on the lower surface of the membrane were fixed in methanol and stained with DAPI. (**A**) Graph representing significant (*P<0.01) decrease in migration of mutant keratincoytes in presence of MMC compared to the wild type, at days 2 and 4 after scratching. The width of the cell free area was measured and the calculated migration distance was plotted against days. The values are mean ± SEM. (**B**) Graphical representation of motility of Ctip2 knockout keratinocytes presented as percent of wild type cells determined by transwell assay.
(EPS)

Figure S3 **Characterization of adult Ctip2^ep−/−^ mice skin and expression of stem cell markers.** (**A**) Immunostaining of Ctip2 (red) in 4 weeks old Ctip2^ep−/−^ mice dorsal skin. (**B**) Immunoblot analyses of Ctip2 protein expression in Ctip2^L2/L2^ and Ctip2^ep−/−^mice dorsal skin. β-actin is used as an internal control. (**C**) Immunostaining for BrdU (green) and Ki-67 (in red) in skin from Ctip2^L2/L2^ and Ctip2^ep−/−^ adult mice. (**D**) Quantification of epidermal BrdU and Ki-67 positive cells in the skin did not reveal significant difference between wild type and control (indicated by # P>0.05). (**E**) Quantification of epidermal thickness. Significant (*P<0.05) increase in epidermal thickness in Ctip2**^ep−/−^** compared to Ctip2^L2/L2^ mice. (**F**) Immunostaining for Keratin 14 (red) on Ctip2^L2/L2^ and Ctip2^ep−/−^ adult mice skin. All sections (A, C and E) were counterstained with DAPI (blue). Yellow dotted yellow lines demarcate the epidermis from dermis (A). E- epidermis, D- dermis. HF-Hair follicle. Scale bar: 50 µm (A-C-D).
(EPS)

Figure S4 **Quantification of proliferation, differentiation and kertainocyte activation and stem cell markers in control and mutant skin.** (**A**) Protein levels of PCNA, K10, K6, K16 and K15 were quantified by densitometric analyses of western blots performed on control and mutant skin at days 3, 5, 7, 9 and 11 post wounding. All data were normalized for ß-actin levels. (**B**) Quantification of the protein level of stem cell markers, Lrig1 and NFATc1 at days 5,9 and 11 post wounding as determined by densitometry analyses of Western blots. All data was normalized by the corresponding ß-actin levels.
(EPS)
